# Molecular and Cellular Mechanisms of Cardioplegic Protection in Surgical Myocardial Revascularization

**DOI:** 10.3390/cells15020173

**Published:** 2026-01-18

**Authors:** Dejan M. Lazović, Milica Karadžić Kočica, Dragan Ivanišević, Vojkan Aleksić, Mladen J. Kočica, Danko Grujić, Jovana M. Mihajlović, Dragan Cvetković, Stefan A. Juričić

**Affiliations:** 1 Clinic for Cardiac Surgery, University Clinical Center of Serbia, 8th Kosta Todorović St., 11000 Belgrade, Serbia; draganivanisevic0503@gmail.com (D.I.);; 2 Faculty of Medicine, University of Belgrade, 11000 Belgrade, Serbia; 3 Center for Anesthesiology, Reanimatology and Intensive Care Medicine, University Clinical Center of Serbia, 8th Kosta Todorović St., 11000 Belgrade, Serbia; 4 Clinic for Cardiology, University Clinical Center of Serbia, 8th Kosta Todorović St., 11000 Belgrade, Serbia

**Keywords:** myocardial ischemia, ischemia–reperfusion injury, oxidative stress, mitochondrial dysfunction, apoptosis, cardioplegia, CABG

## Abstract

**Highlights:**

**What are the main findings?**

- Cardioplegia induces active molecular and cellular myocardial protection by regulating calcium handling, mitochondrial function, oxidative stress, and inflammatory pathways during cardiac arrest.
- Controlled cardioplegic arrest prolongs myocardial ischemic tolerance by suppressing electromechanical activity and reducing metabolic demand.

**What are the implications of the main findings?**

- Mechanistic insights into cardioplegic protection support refinement of cardioplegia composition, temperature, and delivery strategies in CABG surgery.
- Optimization of cardioplegic protocols may improve postoperative myocardial recovery and reduce ischemia–reperfusion-related complications.

**Abstract:**

Coronary artery bypass grafting (CABG) remains the gold standard for patients with advanced multivessel coronary artery disease. Optimal myocardial protection versus ischemia during reversible and controlled cardiac arrest is a cornerstone of successful outcomes. Myocardial ischemia represents a state of reduced coronary perfusion with oxygenated blood, insufficient to meet the metabolic demands of the myocardium. Conventional cardioplegic solutions offer controlled and reversible cardiac arrest while actively modulating the molecular and cellular mechanisms that mediate ischemia–reperfusion injury. Cardioplegia dramatically elongates the reversible period of ischemic injury and restricts cardiomyocyte death by shutting down electromechanical activity, lowering metabolic demand, stabilizing ionic homeostasis, protecting mitochondrial integrity, and slowing oxidative stress and inflammatory signaling. During ischemia, cardiomyocytes shift from aerobic to anaerobic metabolism, resulting in adenosine triphosphate (ATP) depletion, loss of ionic homeostasis and calcium overload that activate proteases, phospholipases and membrane damage. Reperfusion restores oxygen supply and prevents irreversible necrosis but paradoxically initiates additional injury in marginally viable myocardium. The reoxygenation phase induces excessive production of reactive oxygen species (ROS), endothelial dysfunction and a strong inflammatory response mediated by neutrophils, platelets and cytokines. Mitochondrial dysfunction and opening of the mitochondrial permeability transition pore (mPTP) further amplify oxidative stress and inflammation, and trigger apoptosis and necroptosis. Understanding these intertwined cellular and molecular mechanisms remains essential for identifying novel therapeutic targets aimed at reducing reperfusion injury and improving myocardial recovery after ischemic events, particularly in coronary surgery.

## 1. Introduction

Cardioplegia represents both a technique and a procedure of temporarily pharmacologically induced cardiac arrest that enables surgical myocardial revascularization (CABG) to be performed under bloodless and motionless operative conditions. Since its introduction in the mid-20th century, the concept of cardioplegic myocardial protection has evolved from simple electrolyte-based solutions to highly sophisticated formulations designed to modulate specific metabolic and intracellular signaling pathways. The primary objective of cardioplegia is to preserve cellular integrity throughout the ischemic period and to minimize reperfusion injury that occurs upon restoration of coronary blood flow [1]. Cardioplegia is a cornerstone of modern cardiac surgery, allowing safe and controlled myocardial arrest in coronary artery bypass grafting. Despite the fact that cardioplegic arrest does not eliminate ischemia, its effects modify myocardial response to ischemia very markedly by suppressing electromechanical activity and thus reducing oxygen usage [2].

During ischemia, cardiomyocytes are subjected to a range of metabolic and ionic disorders, including ATP depletion, proton and lactate accumulation, mitochondrial dysfunction, and disturbances of calcium homeostasis [3]. Myocardial injury during ischemia is critically dependent on both the duration and severity of oxygen deprivation. Ischemic periods are occasionally reversible, versus long-term ischemia that contributes to irreversible structural and metabolic impact. In the reversible phase of ischemic injury, cardioplegia plays a pivotal role by decreasing ATP use, decreasing calcium overload, improving cell membrane stabilization and protection of mitochondrial function. Nevertheless, irreversible damage may still develop when ischemia is excessive or prolonged emphasizing the significance of a suitable technique of cardioplegics and a controlled reperfusion [4,5].

Although reperfusion is essential for restoring tissue oxygenation levels, whether through grafts or native coronary circulation, it can paradoxically exacerbate further damage to the cell by creating reactive oxygen species (ROS), activating inflammatory signaling cascades, and the initiation of apoptotic pathways [6,7].

A variety of cardioplegic formulations have been developed with the aim of optimizing biochemical conditions during cardiac arrest, reducing energy consumption, and preserving mitochondrial function. Classic crystalloid cardioplegic solutions, such as St. Thomas’s and Bretschneider’s formulations, established the foundational principles for understanding physiological control of electrolytes and pH values, while modern blood-based and modified solutions integrate metabolic substrates, antioxidants, and pharmacological modulating agents [8,9,10].

Given the complexity of molecular interactions, modern research increasingly focuses on elucidating the signaling networks governing cardiomyocyte survival. These include pathways regulating calcium dynamics, oxidative stress, inflammation, and apoptosis. The goal of this review is to synthesize existing evidence on the molecular and cellular mechanisms underlying the actions of cardioplegic solutions, and to highlight emerging experimental and clinical strategies for myocardial protection during coronary surgery.

In this narrative review, there was a search of the literature on the PubMed and Google Scholar databases. Our search strategy consisted of the following keywords and their combinations: ‘cardioplegia’, ‘myocardial protection’, ‘ischemia–reperfusion injury’, ‘coronary artery bypass grafting’, ‘mitochondrial dysfunction’, ‘oxidative stress’, ‘calcium homeostasis’, ‘apoptosis’ and ‘necrosis’. Only peer-reviewed articles published in English were included, with priority given to experimental, translational, and clinical studies published within the last ten years. The reference lists of relevant articles were also screened to identify additional key publication (Appendix A).

## 2. Physiological Basis of Myocardial Ischemia and Reperfusion

Myocardial ischemia is defined as a state of reduced coronary blood flow and oxygen delivery to the heart muscle, resulting in a metabolic imbalance of energy supply and demand. During ischemia, cardiomyocytes shift from aerobic to anaerobic glucose metabolism, leading to decreased ATP synthesis and an increase in lactate and H^+^ concentrations [11]. These alterations cause a decline in intracellular pH and also functional inactivation of key enzymes involved in oxidative phosphorylation.

Importantly, the majority of myocardial injuries do not occur during ischemia itself but during the reperfusion phase. Reperfusion injury is characterized by three main processes: (1) sudden surge in mitochondrial production of ROS, (2) disruption of calcium homeostasis, and (3) activation of inflammatory mediators [12]. ROS damage lipids, proteins, and nucleic acids, while calcium overload promotes opening of mitochondrial permeability transition pores (mPTP), initiating cellular death via necrosis or apoptosis [13,14].

Cardioplegia aims to induce reversible cardiac arrest while preserving cellular structure and metabolic stability throughout the ischemic period (Table 1). 

Its fundamental principles include the following:

1. Chemical induction of asystole—high potassium concentrations (20–30 mmol/L) depolarize the cell membrane and inhibit Na^+^ channel activity, thereby terminating mechanical contraction [15]. Alternatively, hyperpolarizing formulations use agents like lidocaine, Mg^2+^ and low concentrations of K^+^ to suppress the electrophysiological activity of the cell. [16].
2. Metabolic suppression—lowering temperature (4–10 °C) lowers ATP usage up to 80–90%, while blood-based cardioplegia additionally provides a limited supply of oxygen and nutrient substrates [17].
3. Maintenance of ionic and pH homeostasis—includes usage of buffers (buffering agents such as histidine, bicarbonates) and Mg^2+^ to help stabilize the membrane and the amino acids/glucose, supporting energy conservation [18].
4. Controlled reperfusion—gradual reintroduction of cardioplegic solutions as well as oxygen prevents abrupt ROS generation and facilitates ATP replenishment (the Buckberg “hot shot” terminal dose of warm blood cardioplegia). Additives such as mannitol and ascorbate reduce oxidative stress during this critical phase [19].

Effective cardioplegia relies on the synergy of these principles and on precise optimization of electrolyte composition, temperature, and pharmacologic additives.

## 3. Molecular and Cellular Mechanisms of Myocardial Protection

Cardioplegic strategies work by the action of various molecular and cellular mechanisms on myocardial protection. Suppression of electrical activity reduces calcium influx via voltage-dependent channels, and magnesium and sodium ion-channel blockers also help to moderate calcium overload. Stabilization of intracellular calcium homeostasis prevents activation of calcium-dependent proteases and mitochondrial permeability transition pore opening. Also, cardioplegia stabilizes mitochondrial membranes and preserves oxidative phosphorylation and limits excessive generation of reactive oxygen species during reperfusion [20,21].

Cardioplegia exerts its protective effects by influencing several cellular and molecular key mechanisms that determine myocardial resilience to ischemia–reperfusion injury (Table 2 and Table 3):

### 3.1. Calcium Homeostasis

Ca^2+^ influx overload during ischemia and especially during the reperfusion stage activates calpains, phospholipases, and endonucleases, leading to degradation of the contractile apparatus and structural components of cardiomyocytes [22]. Cardioplegia blocks Ca^2+^ entry through membrane depolarization induced by K^+^ concentrations, competitive inhibition of Ca^2+^ channels with Mg^2+^, blockage of Na^+^ channels using lidocaine (Del Nido) [23]. By preventing Ca^2+^ overload, cardioplegia limits the opening of the mPTP and promotes cellular survival and recovery.

### 3.2. Oxidative Stress and Antioxidant Defense

Reperfusion in the aftermath of ischemia consists of a series of acute events characterized by a burst production of reactive oxygen species, activation of inflammatory response cascades and the onset of programmed cell death. Antioxidant, nitric oxide and anti-inflammatory agents in cardioplegic solutions have anti-oxidative activity and, inhibit pro-apoptotic signaling pathways as well as reduce leukocyte activation. Taken together, these effects restrict myocardial stunning, necrosis, and postoperative contractile dysfunction [21].

Reperfusion is associated with a steep increase in ROS production: O_2_^−^, H_2_O_2_, •OH [24]. Antioxidative mechanisms of cardioplegia include mannitol, glutathione and ascorbate, which help maintain NADH/NAD^+^ i GSH/GSSG balance [25]. ROS activate NF-κB i MAPK signaling pathways, which in return enhance expression of proapoptotic proteins; antioxidant-enriched cardioplegia attenuates these pathways and limits subsequent tissue injury [26].

### 3.3. Mitochondrial Function

Cardioplegia stabilizes mitochondrial membranes and preserves the function of respiratory chain complexes I and IV; in addition, adenosine and glutamate enhance ATP synthesis, support mitochondrial integrity, and contribute to more efficient energy recovery during reperfusion [27,28,29]. Ischemia severely inhibits oxidative phosphorylation, causing rapid ATP depletion and metabolic acidosis. Cardioplegic arrest reduces myocardial energy consumption by up to 90% through suppression of electromechanical activity. Substrate-enriched cardioplegic solutions provide glucose, amino acids, and buffering agents that support residual ATP production and stabilize intracellular pH. Preservation of mitochondrial integrity during cardioplegic arrest facilitates rapid recovery of aerobic metabolism upon reperfusion [30,31].

### 3.4. Apoptosis and Inflammation

Apoptosis: during ischemia–reperfusion, activation of caspases 9 and 3 follows cytochrome c release from mitochondria [32].

Cardioplegia counteracts these pathways by increasing Bcl-2, reducing Bax expression, and activating prosurvival kinases such as AKT/ERK pathways [33].

Inflammation lowers TNF-α i IL-6 expression, inhibiting ICAM-1 i NF-κB utilizing Mg^2+^, lidokaine i NO-donors [34].

### 3.5. Energetic Metabolism and ATP

Ischemia impairs oxidative phosphorylation, leading to rapid ATP depletion. Cardioplegia slows metabolic activity and preserves ATP stores, thereby maintaining cellular viability until reperfusion is established.

## 4. Classification of Cardioplegic Solutions

**Figure 1:**
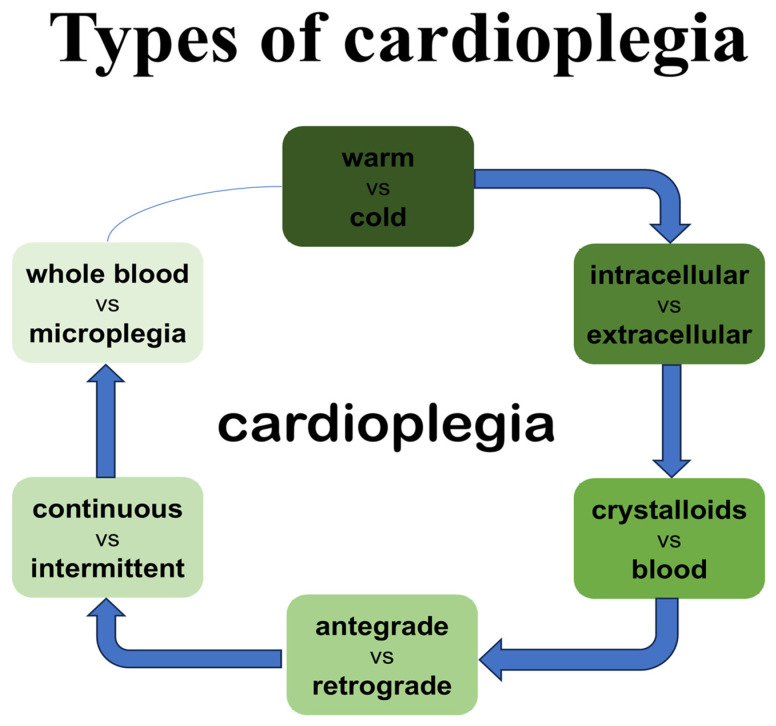
Types of cardioplegia solution.

Cardioplegic solutions may be classified according to their components, mechanism of inducing cardiac arrest, and temperature of administration as well as the way of application (Figure 1).

### 4.1. Classification by Composition

(a) Crystalloid cardioplegia:
  - “Extracellular-type solutions” (which approximate the electrolyte composition of serum) and
  - “Intracellular-type solutions” (which mimic the intracellular electrolyte environment);
(b) Blood cardioplegia:
  Blood-based strategies utilize the biochemical and rheological advantages of oxygenated blood.
  - Standard blood cardioplegia involves mixing crystalloid cardioplegia with blood during administration in various and adequate proportions (cardioplegia: blood = 1:4, 1:8);
  - Microplegia ensures that pure oxygenated normothermic blood is being used with added agents to stop the heart, but also with other cardioprotective additives, to avoid hemodilution and thus enhances the physiologic benefits of blood as a delivery medium.

### 4.2. Classification by Mechanism of Inducing Cardiac Arrest (i.e., Preventing Action Potential Conduction)

(a) Depolarizing cardioplegia

By depolarization of the cell membrane of the myocyte, the resting membrane potential (−80 mV) becomes less negative.

Depolarizing cardiac arrest is achieved by elevating extracellular K^+^ concentration, which in the initial phase should be high (20–30 mM/L) for rapid induction of heart arrest, followed by a maintenance concentration that is brought down to 10 mM/L, which is enough to sustain asystole. This method is well established, reliable, and extensively validated through years of various experimental and clinical studies, as well in practice. However, hyperpotassium-induced arrest has several drawbacks. Prolonged depolarization causes coronary vasoconstriction, accumulation of Na^+^ and cellular edema, endothelial injury, activation of slow Ca^2+^ channels with consequent Ca^2+^ accumulation, and depletion of intracellular ATP, all of which contribute to reperfusion injury, and thus ultimately in significant effect to postoperative arrhythmias and contractile dysfunction of the myocardium [35,36];

(b) Hyperpolarizing cardioplegia

**Figure 2:**
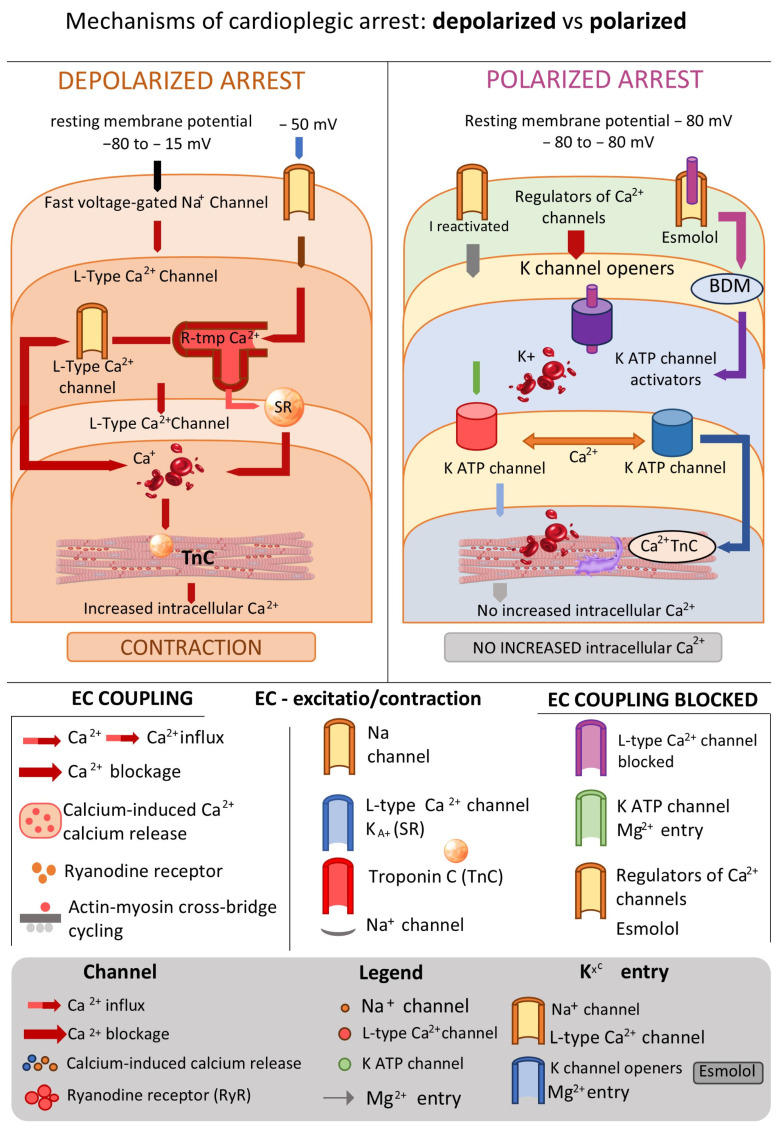
Mechanisms of depolarized and polarized cardioplegic arrest and their effects on excitation–contraction coupling.

By hyperpolarization of cellular membrane of the cardiomiocytes, the resting potential becomes more negative and this can be achieved through various mechanisms, including the following: blocking Na^+^ channels (e.g., lidocaine) which alone is insufficient for safe arrest at non-toxic doses, but is being used as a valuable adjunct in lower concentrations ~0.3 mM/L; opening KATP^+^ kanala (e.g., adenosine) that also cannot on its own produce safe arrest due to short half-life (10 s), thus serving primarily as a supplemental additive in cardioplegias; reducing extracellular Na^+^ concentration, which represents the basis of Bretschneider‘s cardioplegia. Hyperpolarizing heart arrest resembles a state of “physiological hibernation” of the myocardium, as the membrane potential remains close to its native resting level. This prohibits the opening of voltage-dependent ion channels: fast Na^+^ channels responsible for initiating the phase of the action potential, thus preventing heart contraction, slow Ca^2+^ channels, and changing channels Na^+^/H^+^ i Na^+^/Ca^2+^, thus preventing intracellular Ca^2+^ accumulation, vasoconstriction, endothelial damage, cellular edema, decreased consumption of ATP. Clinically, all this leads to reducing postoperative myocardial contractile dysfunctions and arrhythmias. This form of arrest may provide superior cardioprotection under normothermic conditions [37] (Figure 2).

Cardiomyocytes keep the polarization on membrane potential of approximately −80 mV in physiological conditions. Electrophysical stimulation leads to the opening of rapid voltage-gated sodium (Na^+^) channels, forming an action potential, which is coupled with calcium (Ca^2+^) influx through L-type Ca^2+^ channels. Ca^2+^ entry triggers calcium-induced calcium release from the sarcoplasmic reticulum through ryanodine receptors and leads to the ↑ concentration of Ca^2+^ in the cytosol and subsequent binding of Ca^2+^ to troponin C that in turn favors actin–myosin cross-bridge cycling and myocardial contraction. In depolarized cardioplegic arrest, high extracellular potassium (K^+^) levels (>15 mM) decrease the transmembrane K^+^ gradient and partially depolarize the membrane to approximately −50 mV. The extended depolarization resulted in the inactivation of quick Na^+^ channels, consequently preventing action potential propagation and electrical excitation. Nevertheless, incomplete depolarization can enable residual Ca^2+^ influx, resulting in an increased intracellular Ca^2+^ burden, diastolic tension and possibly calcium-overload-induced myocardial damage. In contrast, polarized cardioplegic arrest seeks to maintain the membrane potential near its physiological resting state. This is accomplished via Na^+^ channel blockers, Ca^2+^ channel regulators, potassium channel openers (e.g., K_ATP channel activators), adenosine through AT1 receptor pathways, and agents, e.g., magnesium. While membrane polarization is maintained, voltage-dependent Ca^2+^ channels become closed, intracellular Ca^2+^ levels are lowered, and excitation–contraction coupling is efficiently suppressed without initial depolarization. In addition, a direct inhibition of myofilament activity (e.g., by 2,3-butanedione monoxime, BDM) further reduces contractile activation and energy consumption. In total, cardioplegic strategies disrupt myocardial excitation and contraction through inhibition of electrical and downstream calcium-dependent contractile mechanisms, thereby decreasing myocardial oxygen consumption and protecting the heart during ischemic arrest.

### 4.3. Classification by Temperature: Cold (4–10 °C), Tepid (27–30 °C) and Warm (37 °C) Cardioplegia

Hypothermia significantly reduces metabolic activity of the myocardium, lowers ROS formation, and decreases Ca^2+^ accumulation while maintaining endothelial and microvascular flow and integrity. Myocardial hypothermia has been shown to attenuate ischemia–reperfusion injury and improve post-ischemic functional recovery of the myocardium in the service of cardioprotection [38,39,40].

The perfusion method is highly effective in achieving myocardial hypothermia. Cold cardioplegia is typically re-administered every 20 min to maintain target myocardial temperatures of 10–15 °C. However, coronary disease, native collateral flow, and heart contact with nearby anatomical structures (such as the descending aorta) may interfere with appropriate cooling of the myocardium, so additional topical cooling of the heart (irrigation with cold fluid, ice slush, or gauze around the heart) and left ventricular venting are commonly used to further control desired temperatures. Hypothermia can have detrimental effects on the myocardium, including cellular edema, disruption of cell membranes, and impaired receptor function on which pharmacotherapy depends. These changes may delay postoperative functional recovery of the myocardium. Normothermic cardioplegia (37 °C) avoids the adverse effects of hypothermia; however, when administered intermittently, it does not provide adequate protection of the myocardium during ischemia [41,42].

Hypothermia is not strictly required for myocardial protection because the majority of cardiac metabolic demands (80–90%) stem from electromechanical activity; once the heart is at arrest, energy consumption decreases substantially. Although hypothermia further reduces metabolic demand by 5–7%, the reduction achieved by electrical arrest alone is sufficient so that only minimal blood flow is necessary to meet residual requirements.

Normothermic blood-based cardioplegia perfuses the heart during ischemia with oxygenated blood that now uses aerobic metabolism that promotes reparative processes and thus avoids ischemia–reperfusion injury while also avoiding the negative consequences of hypothermia.

Theoretically, this may provide better myocardial protection and potentially allow for longer aortic cross-clamp times. In clinical practice, this strategy poses several challenges. Perfusion must be continuous, and with anterograde delivery the surgical field may become obscured due to ischemic congestion, requiring periodic interruption of cardioplegia (up to 15 min). These interruptions may result in cumulative ischemic myocardial injury. Additionally, patients with coronary artery disease may experience inadequate distribution of blood cardioplegia, further predisposing the myocardium to ischemia. Retrograde perfusion can partially compensate for these limitations, but remains unreliable due to the presence of venous shunting, which can be significant. Hypertrophic myocardium, with its increased metabolic demands, is especially vulnerable to ischemic intervals. Continuous application of cardioplegia can also lead to hyperkalemia.

Normothermic cardiopulmonary bypass (CPB) is associated with systemic vasodilatation that makes it difficult to maintain adequate perfusion pressure and often requires vasoconstrictor support. The brain is more susceptible to ischemic injury under normothermic conditions, increasing the risk of neurological complications [43,44].

Several randomized studies have not demonstrated a significant advantage of either of the cardioplegia techniques [45]. A potential solution may involve the combination of different temperatures or the use of mild hypothermia. Hayashida has demonstrated in his works that mildly cold blood cardioplegia (29 °C) combined with mild systemic hypothermia (32 °C) may offer superior cardioprotection compared to warm or profoundly cold blood-based cardiplegia. This approach adequately reduces metabolic requirements while promoting rapid restoration of heart function, and conferring neuroprotective effects [46,47].

A combined strategy may therefore be beneficial, applying warm blood cardioplegia for induction, followed by intermittent cold blood cardioplegia for maintenance, thereby maximizing the benefits and minimizing the limitations of different temperature techniques [39].

### 4.4. Classification by Route of Administration

Cardioplegia may be administered anterograde or retrograde, or through a combined (anterograde-retrograde) approach, delivered either simultaneously or intermittently. In the United States, 60% of surgeons use the combined technique.

- Anterograde—via aortic root, or through a venous graft directly into the coronary ostium;
- Retrograde—via coronary sinus.

Studies have shown that the safest duration of aortic cross-clamp time is up to 4 h with well-balanced cardioplegia distribution, in comparison to 30 min when there are territories distal to coronary stenoses not reachable by cardioplegia resulting in disproportionate distribution of such. For cardioplegia to be effective, it must reach all myocardial regions in sufficient volume. This can be challenging with anterograde delivery in patients with hypertrophic myocardium or severe coronary artery disease, particularly in those undergoing total arterial revascularization, where direct infusion of cardioplegia through venous grafts is not possible. Retrograde cardioplegia overcomes many of these limitations and enhances subendocardial perfusion, which is significantly important in hypertrophic myocardium.

Although most venous drainage from the myocardium enters right atrium via the coronary sinus, a portion of the cardial veins drains through alternative pathways such as veno-venous shunts, the anterior cardiac vein, or Thebesian veins directly into the ventricles—especially the right side of the heart. For this reason, nutritive flow is reduced by retrograde cardioplegia to 30–80% of cardioplegia flow on account of right ventricle (in smaller part and posterior wall of left ventricle) and due to cardioplegic hypoperfusion of the right heart it does not provide optimal cardioprotection. On the other hand, anterograde flow is 90% nutritive and allows for better perfusion and protection of the right ventricle [48,49].

The characteristics of anterograde cardioplegia are as follows:

- Most commonly used technique due to its rapid administration, prompt induction of cardiac arrest, and overall effectiveness.
- Administered intermittently via the aortic root, through venous grafts, or directly into the coronary ostia. Direct coronary perfusion carries a risk of intimal injury of coronary arteries, or “jet” endothelial lesions with generating acute embolization by atherosclerotic debris, or dissection, potentially leading to subsequent ostial stenosis.
- Perfusion pressure of 70–100 mmHg allows uniform distribution of cardioplegia without the risk of endothelial damage nor myocardial edema. Higher pressure may be required in patients with severe coronary disease (130 mmHg); during reperfusion stage, lower perfusion pressure (≤50 mmHg) is recommended.
- Occclusive changes in coronary arteries or hyperthrophic myocardium may impede balanced perfusion and compromise adequate protection of the left ventricle. In patients with “left main” stenosis, when the internal thoracic artery is used, the basin of the anterior left descending artery can remain unprotected due to malperfusion of cardioplegia.
- Left ventricular dilatation is a particular risk in patients with aortic insufficiency, therefore venting of the left chamber is mandatory [50,51,52,53].

The characteristics of retrograde cardioplegia are as follows:

- Indicated in patients with severe coronary disease, “left main” stenosis, aortic regurgitation and hypertrophy of the myocardium (e.g., mitral valve disease, aortic stenosis). It allows more homogeneous and better subendocardial perfusion of the left ventricle without its distention. In this way, direct coronary ostial manipulation is able to be avoided.
- Administered via the coronary sinus, retrograde cardioplegia offers improved visualization of coronary anastomoses compared with anteriograde delivery, allowing continuous cardioplegic perfusion in normothermic cardioplegia, while cold cardioplegia is usually applied intermittently.
- Can be administrated intermittently or simultaneously with anterograde perfusion. Canulation of the coronary sinus can be technically demanding with possible malposition of catheter during the procedure.
- Perfusion pressure must be ≤50 mmHg to minimize risk of coronary sinus rupture, venous injury, perivascular bleeding and myocardial edema. Perfusion pressure bellow <40 mmHg indicates reduced subendocardial perfusion [48,50,51,52,53].

## 5. Cardioplegia: Components and Metabolic Support

**Figure 3:**
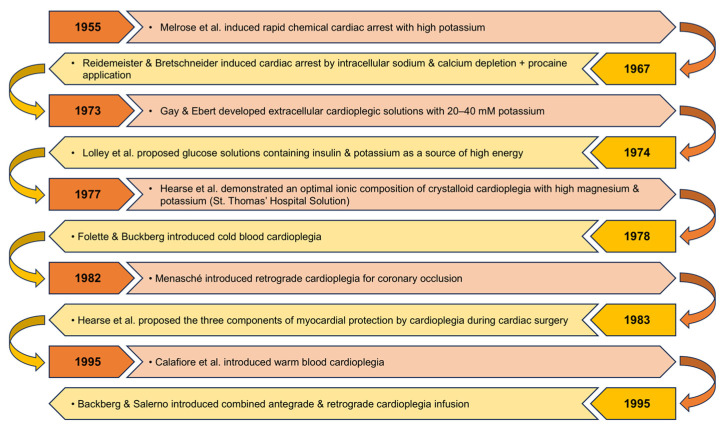
Timeline of the key developments in the history of cardioplegia [57,58,59,60,61,62,63,64,65,66].

In cardioplegia, hypothermia, buffers and energetic substrates (glucose, glutamate, aspartate) help maintain ATP levels and pump function during ischemia [54,55]. Del Nido i Custodiol solutions provide metabolic substrates that prolong ischemic tolerance [56] (Figure 3).

Types of Cardioplegic Solutions: Mechanism and Usage.

Cardioplegic solutions can be categorized among themselves based on their composition, mechanism of action, and molecular effects. Understanding these differences is essential for optimizing myocardial protection during coronary surgery (Table 4 and Table 5).

### 5.1. Cristalloid Solutions

Crystalloid solutions are particularly useful in long, continuous procedures, but often require larger volumes, potentially causing hemodilution and myocardial edema.

St. Thomas I and II and Bretschneider (Custodiol) are widely used crystalloid solutions.

St. Thomas solutions are characterized by the following:

- high concentration of K^+^ (15–30 mmol/L), Mg^2+^ i Na^+^/Ca^2+^ balans.
- induces depolarizing asystole
- advantages include simple preparation, broad applicability
- limitations include short-term ischemic tolerance (~30–40 min), without additional metabolic protection [67].

Bretschneider/Custodiol solutions are characterized by the following:

- contain histidine as a buffer, mannitol as an osmotic agent, and low concentrations of Na^+^ and Ca^2+^.
- induce hyperpolarization and metabolic suppression
- prolong myocardial ischemic tolerance up to 90–120 min under cold asystole.
- experimental data suggests preservation of mitochondrial membranes and reduced ROS during prolonged period of ischemia [68,69] (Table 6).

### 5.2. Blood-Based Cardioplegia

Blood-based cardioplegia is a solution based on autologous blood of the patient, mixed with electrolytes and pharmacologic additives (K^+^, Mg^2+^, prokain).

- Advantages include the following: provideing oxygen and metabolic substrates, reducing anaerobic glycolysis, contains intrinsic antioxidants and buffering systems.
- Limitations include the following: requires precise preparation and compatibility with perfusion systems.
- Molecular effects include the following: reduces ROS and lipid peroxidation, preserves ATP, and enhances recovery of contractile function [70,71].

### 5.3. Modified and Hybrid Solutions (Del Nido)

Del Nido cardioplegia is an example of modern hybrid formulation, originally developed for pediatric patients, now used in adults.

Components include the following: low concentration of Ca^2+^, high concentration of K^+^, Mg^2+^, lidokaine and mannitol; added glucose and aspartate.

- Advantages include the following: prolonged ischemic tolerance (up to 90–120 min in single use), reduced need for repeated administration, preservation of mitochondrial function and ATP.
- Molecular effects include the following: prevents Ca^2+^ overload and mPTP opening, reduces oxidative stress and inflammation, and activates pro-survival signaling pathways [72,73].

Comparative studies indicate that Del Nido and Custodiol solutions provide longer and more stable cardioprotection, with lower oxidative stress and improved ATP preservation, which is especially valuable in complex and long-lasting operations.

Molecular and clinical implications of various cardioplegic formulations and delivery strategies differ markedly. Crystalloid cardioplegia delivers reliable arrest while also leading to hemodilution and limited metabolic support. Blood-based cardioplegia offers better oxygen delivery, buffering capacity, and antioxidant protection. Hybrid solutions like Del Nido cardioplegia combine prolonged ischemic tolerance with reduced calcium overload and enhanced mitochondrial preservation and therefore are particularly applicable to complex and prolonged procedures [74].

Modern-day research in the academic field of cardioprotection is increasingly focused on elucidating the molecular mechanisms of ischemia–reperfusion injury and developing novel strategies to enhance myocardial protection. Experimental models (including in vitro systems, animal models, and isolated perfusion systems) allow testing of innovative additions and modifications to cardioplegic solutions, such as:

1. Cytoprotective additives
  - Adenosine acts as a vasodilator, inhibits Ca^2+^ influx, and activates pro-survival kinases (AKT, ERK1/2). Experimentally, adenosine reduces infarct size and ROS formation in models of myocardial ischemia [75].
  - Nitric Oxide donors and S-nitrosoglutathione modulate vascular tonus and inflammation, inhibit NF-kB and ICAM-1 activation, and reduce neutrophilic infiltration [76].
  - Peptides and hormones, bradykinin, apelin, and urotensin II, can modulate mitochondrial function and activate anti-apoptotic pathways [77].
2. Pharmacological modifiers
  - Lidocaine and magnesium lower Ca^2+^ overload and stabilize membrane potential.
  - Beta-blockers and inhibitors of Na^+^/H^+^ pump have been experimentally shown to reduce reperfusion injury and ROS production [78].
3. Metabolic and energetic additives
  - Glutamate, aspartate, and glucose support the citric acid cycle and ATP synthesis during ischemia.
  - Ketones and creatine-phosphate experimentally prolong myocardial tolerance to ischemia [79].
4. Nanotechnology and targeted strategies
  - Nano-cardioplegia utilizes nanoparticles to deliver antioxidants or pharmacologic agents directly to mitochondria.
  - Genetically targeted therapy—experimental approaches focus on anti-apoptotic proteins (Bcl-2, HSP70) and oxidative stress inhibitors [80].
5. Translational aspects and clinical significance

Experimental studies facilitate the identification of newer molecular markers of cardioprotection that can be translated into clinical practice. For example, modified Del Nido formulations supplemented with the addition of adenosine and Mg^2+^ have been shown to enhance the postoperative function of the left ventricle. A combination of blood-based cardioplegia with antioxidants and pharmacological additives lowers the cost of postoperative support and decreases the risk of arrhythmias [75,80].

The overarching goal of these strategies is to transform cardioplegia from a passive technique into an active therapeutic intervention, modulating intracellular pathways and optimizing myocardial metabolism.

## 6. Conclusions

Cardioplegic solutions represent a cornerstone of myocardial protection during cardiac surgery, especially in coronary artery procedures. Their protective effect extends beyond the physiological induction of asystole, encompassing a complex network of molecular and cellular mechanisms, including maintenance of Ca homeostasis, regulation of oxidative stress, stabilization of mitochondrial function, modulation of apoptotic and inflammatory pathways, and also preservation of energetic metabolism.

Contemporary strategies of cardioplegia, including modified crystalloid solutions, blood-based cardioplegia, and hybrid formulations such as Del Nido, allow prolonged ischemic tolerance and reduced reperfusion injury. Experimental additions, such as adenosine, NO-donors, antioxidants, and pharmacological modifiers, provide additional enhancement of cellular protection.

A thorough understanding of the molecular and cellular mechanisms underlying myocardial protection forms the foundation for the development of next-generation cardioplegic solutions and personalized strategies for high-risk patients. These approaches optimize postoperative function of the heart and reduce perioperative complications.

From a translational research perspective, the ultimate goal is to shift cardioplegia from a passive protective technique to an active therapeutic approach, enabling precise, targeted modulation of intracellular signaling pathways and metabolic processes.

## Figures and Tables

**Table 1:** Principles and Composition of Cardioplegia.

Principle	Component
Rapid diastolic arrest	K^+^ 20–25 mM/L
Buffers: pH 7.30–7.60 (25 °C)	Histidine, bicarbonates, blood, tromethamine
Reduction in Ca^2+^ level	Hypocalcemia (0.015–0.5 mM/L); Chelates (citrate–phosphate–dextrose)
Homogeneous distribution	Antegrade ± retrograde application
Temperature	Cold vs. moderate vs. warm (4–10 °C vs. 20–32 °C vs. 37 °C)
Cardioprotective additives	O_2_–blood; Mannitol, glucose, albumin; Amino acids (glutamate, aspartate); Glucose + insulin; Mg^2+^; Adenosine; Lidocaine

**Table 2:** Strategies for Cardioplegic Reduction in Ischemic Injury.

Principle	Mechanism
Reduction in O_2_ demand/consumption	Hypothermia; Asystole; Continuous perfusion
Anaerobic metabolism	Blood; Oxygenated crystalloid cardioplegia
Metabolic substrate of the energy cycle	Glucose; Amino acids (glutamate, aspartate)
Correction of acidosis	Hypothermia (Rosenthal effect); Buffers: tromethamine, histidine, bicarbonates, blood
Optimization of metabolism	Induction—warm cardioplegia; Maintenance—cold cardioplegia; Reperfusion—warm cardioplegia; Intermittent application
Reduction in Ca^2+^ accumulation	Hypocalcemia; Chelates (citrates); Antagonists (Mg^2+^); Ca^2+^ channel blockade (adenosine)
Reduction in edema	Hyperosmolarity: mannitol, glucose, KCl; Perfusion pressure: 50 mmHg

**Table 3:** Strategies for Cardioplegic Reduction in Reperfusion Injury.

Principle	Mechanism
O_2_ delivery	Blood (Hct = 0.20–0.25)
Normothermia	36.5–37 °C
Reduction in myocardial metabolism	Initial maintenance of arrest; Empty heart (LV vent)
Prevention of Ca^2+^ accumulation	Hypocalcemia (≤0.5 mmol/L); Chelates (citrates); Antagonists (Mg^2+^); Ca^2+^ channel blockade (adenosine)
Antiedematous effect	Perfusion pressure low (≤50 mmHg); Hyperosmolarity (glucose, mannitol, albumin)
Inhibition of oxygen-free radicals	Perfusion pressure low (≤50 mmHg); Inhibition of production; Superoxide dismutase, xanthine oxidase, catalase; Scavengers (allopurinol, nitric oxide, mannitol)
Antileukocyte effect	Filters for leukocyte depletion; Leukocyte inhibitors (adenosine, nitric oxide, antibodies)
Correction of acidosis	Buffers: tromethamine, histidine, bicarbonates, blood
Metabolic substrates	Amino acids (aspartate, glutamate); Glucose, insulin, potassium

**Table 4:** Time-dependent metabolic and cellular modifications during cardioplegic arrest.

Duration of Cardioplegic Arrest	Metabolic Modifications	Cellular and Subcellular Changes	Functional/Clinical Implications
Short duration	Minimal ATP depletion; preserved phosphocreatine; limited anaerobic glycolysis; stable intracellular pH	Maintained ionic homeostasis; controlled Ca^2+^ flux; preserved mitochondrial integrity	Effective myocardial protection; rapid functional recovery after reperfusion
Intermediate duration	Progressive ATP and phosphocreatine depletion; lactate accumulation; intracellular acidosis	Na^+^/K^+^-ATPase and SERCA dysfunction; intracellular Na^+^ and Ca^2+^ overload; early mitochondrial impairment	Increased risk of myocardial stunning; delayed recovery of contractile function
Prolonged duration	Severe high-energy phosphate depletion; pronounced acidosis; impaired oxidative metabolism	Mitochondrial permeability transition pore opening; loss of membrane potential; activation of apoptotic and necrotic pathways	High risk of irreversible myocardial injury; reduced postoperative ventricular function

**Table 5:** Composition of standard cardioplegic solutions in use—Clinic for Cardiac Surgery, University Clinical Centre of Serbia.

Component (mM/L)	Bretschneider	St. Thomas Hospital	Del Nido (1 Blood: 4 Cardioplegia)	Blood (4 Blood: 1 Cardioplegia, Induction → Maintenance)
Na^+^	15	120	145	144
K^+^	9	16	24	20–25 → 10
Mg^2+^	4	16	7	5
Ca^2+^	0.015	1.2	0.4	0.50 → 0.25
Glucose/Insulin	/	/	1	>0.2/2.5 IU
Citrate-phosphate-dextrose	/	/	/	0.3
Mannitol	30	/	14	60
Lidocaine	/	/	0.36	/
NHCO_3_^−^	/	/	/	/
Histidine	198	10	/	/
Trometamol (THAM)	/	/	/	0.3 M/L
pH (25 °C)	7.20	7.8	7.4	7.6
Tryptophan	2	/	/	/
Ketoglutarate	1	/	/	/
Glutamate/Aspartate	/	/	/	12/12
Osmolarity (mOsm/L)	310	304	375	~380
Hematocrit	/	/	~6	~25

**Table 6:** Comparative characteristics of commonly used cardioplegic solutions.

Type of Cardioplegia	Composition/Characteristics	Advantages	Disadvantages	Clinical Implications
Crystalloid cardioplegia (St. Thomas)	Extracellular-type; high potassium; cold; repeated dosing	Rapid diastolic arrest; simple preparation; wide availability	Repeated administration; myocardial edema; no oxygen delivery	Short cross-clamp times; routine procedures
Crystalloid cardioplegia (Bretschneider—HTK)	Intracellular-type; low sodium/calcium; single-dose; large volume	Prolonged protection; uninterrupted surgical field	Hemodilution; reduced oxygen delivery; less effective in hypertrophied myocardium	Complex or prolonged procedures; caution in CAD
Cold blood cardioplegia	Oxygenated blood with crystalloid; hypothermic	Better oxygenation; buffering capacity; reduced ischemic injury	More complex preparation; repeated dosing	Standard in adult cardiac surgery; ischemic myocardium
Warm blood cardioplegia	Normothermic; intermittent or continuous	Improved post-ischemic recovery; less reperfusion injury	Risk of inadequate cooling; higher metabolic demand	Poor ventricular function with careful delivery
Del Nido cardioplegia	Low calcium; lidocaine; magnesium; single-dose; blood-crystalloid	Prolonged arrest; reduced calcium overload; simple workflow	Limited adult long-term data; redosing strategy unclear	Increasing use in adult CABG and complex surgery

## Data Availability

The original contributions presented in this study are included in the article. Further inquiries can be directed to the corresponding authors. All figures included in manuscript are original and were created by authors. They do not been published anywhere.

## Appendix A. Materials and Methods

Cardioplegic solutions remain an essential component of myocardial protection in CABG surgery. The main objective of this review was to analyze the molecular, cellular, and metabolic mechanisms underlying myocardial ischemia/reperfusion injury.

A comprehensive literature search was conducted using the PubMed and Google Scholar databases, employing the following keywords: myocardial ischemia, reperfusion injury, mitochondrial dysfunction, reactive oxygen species (ROS), apoptosis, and necroptosis.

The analysis included clinical, preclinical, experimental, and review studies that investigated cellular and metabolic processes during ischemia–reperfusion injury, with a particular focus on the mechanisms of oxidative stress, apoptosis, necroptosis, autophagy and mitochondrial dysfunction.

Only articles published in English were included, with the majority of data derived from studies published in the last ten years. Abstracts, non-peer-reviewed papers and unpublished data were excluded from this review.
